# Progressive obesity leads to altered ovarian gene expression in the Lethal Yellow mouse: a microarray study

**DOI:** 10.1186/1757-2215-2-10

**Published:** 2009-08-03

**Authors:** John Brannian, Kathleen Eyster, Mandi Greenway, Cody Henriksen, Kim TeSlaa, Maureen Diggins

**Affiliations:** 1 Department of Obstetrics & Gynecology, Sanford School of Medicine, University of South Dakota, Sioux Falls, SD 57105, USA; 2 Division of Basic Biomedical Sciences Sanford School of Medicine, University of South Dakota, Vermillion, SD, USA; 3 Sanford Research USD, Sioux Falls, SD, USA; 4 Department of Biology, Augustana College, Sioux Falls, SD, USA

## Abstract

**Background:**

Lethal yellow (LY; C57BL/6J *A*^*y*^/*a*) mice exhibit adult-onset obesity, altered metabolic regulation, and early reproductive senescence. The present study was designed to test the hypothesis that obese LY mice possess differences in expression of ovarian genes relative to age-matched lean mice.

**Methods:**

90- and 180-day-old LY and lean black (C57BL/6J *a/a*) mice were suppressed with GnRH antagonist (Antide^®^), then stimulated with 5 IU eCG. cRNA derived from RNA extracts of whole ovarian homogenates collected 36 h post-eCG were run individually on Codelink Mouse Whole Genome Bioarrays (GE Healthcare Life Sciences).

**Results:**

Fifty-two genes showed ≥ 2-fold differential (p < 0.05) expression between 180-day-old obese LY and lean black mice. LY mice exhibited elevated ovarian expression of agouti (350×), leptin (6.5×), and numerous genes involved in cholesterol/lipid transport and metabolism, e.g. lanosterol synthase, *Cyp51*, and steroidogenic acute regulatory protein (*Star*). Fewer genes showed lower expression in LY mice, e.g. angiotensinogen. In contrast, none of these genes showed differential expression in 90-day-old LY and black mice, which are of similar body weight. Interestingly, 180-day-old LY mice had a 2-fold greater expression of 11beta-hydroxysteroid dehydrogenase type 1 (*Hsd11b1*) and a 2-fold lesser expression of 11beta-hydroxysteroid dehydrogenase type 2 (*Hsd11b2*), differences not seen in 90-day-old mice. Consistent with altered *Hsd11b *gene expression, ovarian concentrations of corticosterone (C) were elevated in aging LY mice relative to black mice, but C levels were similar in young LY and black mice.

**Conclusion:**

The data suggest that reproductive dysfunction in aging obese mice is related to modified intraovarian gene expression that is directly related to acquired obesity.

## Background

The negative impact of obesity on fertility is well recognized [1-3]. Moreover, obesity leads to progressive health disorders associated with the metabolic syndrome. These include polycystic ovary syndrome (PCOS), which is the most prevalent endocrinopathy of reproductive age women and a major cause of infertility. Numerous animal models of obesity have been studied, including the *ob/ob *and *db/db *mutant mouse strains. However, these mouse models do not mimic typical human obesity. The *ob/ob *mouse, for example, lacks bioactive leptin [4] whereas the *db/db *mouse possesses a dysfunctional leptin receptor [5]. These types of mutations resulting in complete dysregulation of body weight control are rarely found in the human population.

The lethal yellow (LY) mouse (C57BL/6J *A*^*y*^/*a*) possesses a gene deletion in the promoter and first exon region of the agouti protein gene locus that brings an upstream promoter into place, resulting in the inappropriate constitutive expression of the agouti gene [6]. In the hypothalamus, the over-expressed agouti protein acts as an antagonist of melanocortin-4 receptors (MCR4) [7], which play a critical role in central appetite and metabolism regulation [8]. This interferes with normal satiety control resulting in hyperphagia [9]. As a consequence, LY mice exhibit progressive adult-onset obesity, and gradually develop insulin resistance [10], hyperleptinemia [11,12], central leptin resistance [13].

Early reproductive senescence is also a hallmark feature of the *A*^*y*^/*a *genotype [12,14-18]. Granholm and co-workers [14] found that LY mice over 120 days old exhibited abnormal estrous cyclicity and decreased mating success relative to age-matched black mice lacking the agouti mutation (C57BL/6J *a/a*), although ovulation rate did not differ. Based on vaginal smears, younger (< 120 days) LY mice had estrous cycles of 4–5 days in length and were indistinguishable from cycles of age-matched black mice [15]. With advancing age and progressively increasing obesity, the estrous cycles of yellow mice lengthened and prematurely ceased between 200–250 days [15]. Ovarian function could be maintained in aged LY mice stimulated with eCG/hCG, although fewer developing embryos tended to be recovered than from identically-treated black mice [14].

To elucidate whether impaired fertility in aging LY mice was due to intrinsic ovarian defects or to extraovarian factors, Granholm and Dickens [16] performed reciprocal ovarian transplantation between young (70–90 days old) LY (*A*^*y*^/*a*) and black (*a/a*) mice and followed reproductive function as the animals aged. Black mice with transplanted ovaries from LY mice exhibited normal fertility. In contrast, LY mice with transplanted ovaries from black mice experienced diminished reproductive function similar to intact LY mice [16]. These authors concluded that there was no underlying intrinsic defect in the ovaries of LY mice, but rather impaired fertility must result from either abnormal hypothalamic-pituitary control or from extraovarian factors that altered the function of ovarian cells.

The loss of reproductive function in LY mice is directly related to obesity. LY mice maintained on a fat-restricted diet that kept their body weight under 30 g, continued to cycle normally as they aged, but LY mice weighing more than 30 g acquired irregular and lengthened cycles [17]. In addition, 270-day old LY mice fed a low-fat diet had similar ovarian histology and equivalent number of antral follicles on proestrus as age-matched black mice [18]. Premature cessation of ovulation in aging LY mice correlated with increasing body weight and circulating leptin concentrations [12]. Moreover, *in vitro *blastocyst development of embryos from 180-day LY mice was impaired compared with embryos from black mice, and this correlated negatively with leptin levels [12]. Collectively these results suggest that early loss of fertility in LY mice is the result of progressive obesity, which is mediated by altered ovarian function as the result of either modified gonadotropic control and/or extraovarian factors arising from obesity. The present study was designed to test the hypothesis that progressive obesity in LY mice alters ovarian gene expression independently of altered hypothalamic-pituitary control.

## Methods

### Animals

The study was approved by the Augustana College Animal Care and Use Committee. Black (C57BL/6J *a/a*) and LY (C57BL/6 *A*^*y*^/*a*) mice from the Augustana College Biology Department breeding colony were used for the study. Founder mice were originally obtained from The Jackson Laboratory (Bar Harbor, ME, USA). Mice were fed maintenance diet (Harlan Teklad, Madison, WI, USA) and fresh water *ad libitum*, and housed in groups of three mice per cage on a 14:10 light/dark cycle with lights on at 0600 [12].

To exclude gonadotropin-mediated effects, 90- and 180-day old LY and black female mice were suppressed with GnRH antagonist (Antide^©^, Bachem, Torrance, CA) prior to administration of eCG (Sigma) to stimulate coordinated follicle development. The ovarian suppression protocol was validated in preliminary studies by suppression (> 80%) of serum FSH, cessation of cyclicity based on vaginal cytology, and absence of large follicles and corpora lutea on ovarian histology (unpublished data). Late estrus/metestrus mice were given Antide (10 μg/g BW, i.p.) on the morning of day 1 of treatment, and again on the morning of day 4. On the evening of day 5, mice were injected i.p. with 1 IU/5 g BW eCG. The mice were sacrificed 36 hours after eCG injection and ovaries immediately removed and trimmed of surrounding fat and connective tissue. Ovaries were placed in RNA Later (Ambion, Austin, TX) for subsequent RNA extraction.

### RNA Extraction

RNA was extracted as described [19]. Each ovary was homogenized in 1 ml TRI reagent (Molecular Research Center, Cincinnati, OH). Sodium acetate and bromochloropropane were mixed with the homogenate, the sample was incubated on ice for 15 min, and then centrifuged to separate the phases. The aqueous phase containing RNA was removed and purified on an RNeasy column (Qiagen, Valencia, CA). The sample was treated with an on-column RNase-free DNase to remove any potentially contaminating genomic DNA. Total RNA was eluted from the column. The RNA concentration and purity were calculated using the RNA 6000 Nano LabChip in an Agilent Bioanalyzer. The RNA was stored at -70°C prior to processing for DNA microarray analysis.

### DNA Microarrays

CodeLink Whole Mouse Genome Bioarrays (GE/Amersham, Piscataway, NJ, now Applied Microarrays, Tempe, AZ) were used for the analysis of differential gene expression. These microarrays contain 3.3 × 10^4 ^single-stranded 30-mer oligonucleotide probes for mouse genes and transcribed sequences. Biotinylated cRNA probes were synthesized from the extracted RNA samples per supplier's directions as previously described [19] using CodeLink Expression Assay Reagent Kit (GE-Amersham Biosciences). Individual samples were run on separate microarrays (90-day LY n = 3, 90-day black n = 3, 180-day LY n = 3, 180-day black n = 3); no samples were pooled. The biotinylated cRNA was fragmented and hybridized with the DNA microarray slides for 18 hours at 37°C. The hybridized slides were washed and incubated with streptavidin-Alexa Fluor 647 (Molecular Probes/Invitrogen) to label the cRNA and washed again. An Axon GenePix Scanner was used to scan the microarrays. GenePix Pro software (MDS, Inc., Toronto, ON) was used to acquire and align the microarray image. CodeLink software (Applied Microarrays, Tempe, AZ) applied the background correction. GeneSpring 7.0 software (Agilent, Santa Clara, CA) was used to normalize the expression of each gene to the median gene expression and to normalize each slide to the 50^th ^percentile of gene expression. Statistical analysis of the data was performed using GeneSpring 7.0 (Agilent), with the p value set at 0.05 for the t-test. Multiple testing correction used the Benjamini and Hochberg False Discovery Rate. Approximately 5% of the genes would be expected to pass this restriction by chance with this test. The data set for these DNA microarrays has been deposited at the National Center for Biotechnology Information Gene Expression Omnibus [GEO; ] as recommended by Minimum Information About a Microarray Experiment [MIAME] standards and can be accessed through accession number GSE14937.

### Real Time RT-PCR

Pre-designed primers and fluorescent (FAM) labeled minor groove binding probe were obtained from Applied Biosystems (Foster City, CA). Real time RT-PCR was carried out with TaqMan Gold RT-PCR reagents (Applied Biosystems) as described [19]. Changes in expression of genes of interest were calculated relative to an endogenous control (GAPDH). An RNA concentration-response validation curve was carried out to determine the concentration of RNA to add to the RT-PCR reaction. All samples were run in duplicate, n = 3 animals. The Relative Expression Software Tool (REST^©^) [20] was used to analyze the data from the real time RT-PCR reaction.

### Radioimmunoassay and Tissue Extraction for Corticosterone Measurement

An additional set of 90- and 180-day old LY and black mice (n = 5 per group) was GnRH antagonist-suppressed and eCG-stimulated as described earlier. Both trimmed ovaries from each animal were combined, weighed and homogenized in a 200 μL of methanol to extract the steroids, yielding ~90% recovery efficiency. Corticosterone concentrations in ovarian extracts were measured using a competitive RIA for mouse and rat corticosterone (MP Biomedicals, Orangeburg, NY). All samples were run in a single assay run. Intra-assay CV was ~7.5%. Tissue concentrations were expressed as ng/mg wet weight, and were compared among groups by ANOVA with Fisher's LSD test.

## Results

**Figure 1:**
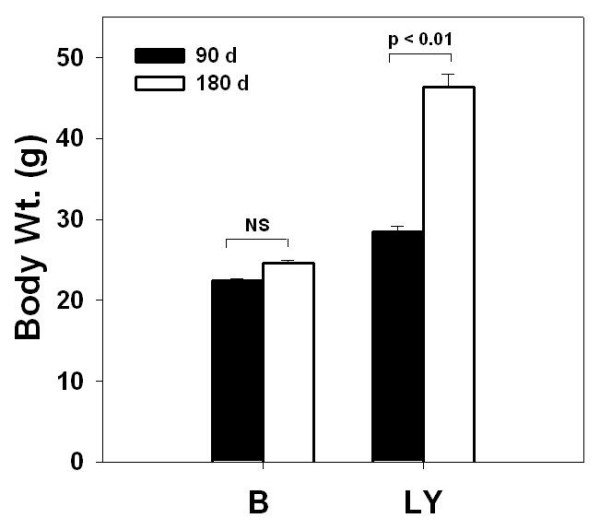
**Body weight (g) of 90 and 180-day black and LY mice (mean ± SEM; n = 3 for each group)**.

There was no difference in body weight between 90-day old LY and black mice, but 180-day old LY mice were significantly heavier than black mice (Figure 1). Initial DNA microarray experiments were conducted using 180-day old mice to determine whether there were differences in ovarian gene expression between obese LY mice and lean black mice. Unidentified genes and expressed sequence tags (EST) were removed from analysis, as were those genes whose expression was less than 0.2 relative intensity units (the limit of sensitivity) in both control and treatment groups. To limit analysis to those genes most likely to be physiologically relevant, only those identified genes with at least a 2 ± 0.1-fold difference in expression were included in the final data set. After these exclusions, 52 of the roughly 3.3 × 10^4 ^genes analyzed with the microarrays showed statistically significant differential expression. Twenty-eight of the differentially expressed genes have indentified protein products (Table 1). Two of these genes, agouti and *Raly *(hnRNP-associated with lethal yellow), a gene located in the deleted segment responsible for the LY syndrome, exhibited the expected differences in relative expression, i.e. agouti was 350-fold greater in LY mice and *Raly *expression was half that in black mice.

**Table 1:** Genes with differential (2.0 ± 0.1-fold; p < 0.05) ovarian expression in 180-day LY mice compared to age-matched black mice.

**Accession Number**	**Relative Expression**	**Name**
NM_028744.1	0.4	phosphatidylinositol 4-kinase type 2 beta
AK041828.1	0.4	SH3-domain kinase binding protein 1
*NM_023130.1*	0.5	**hnRNP-associated with lethal yellow (*Raly*)**
NM_018867.3	0.5	carboxypeptidase × 2 (M14 family)
NM_008289.1	0.5	hydroxysteroid 11-beta dehydrogenase 2 (*Hsd11b2*)
AW411692.1	0.55	BCL2-like 11 (apoptosis facilitator)
NM_010350.1	0.55	glutamate receptor, ionotropic, NMDA2C (epsilon 3)
NM_007428.2	0.55	angiotensinogen
		
NM_009338.1	1.9	acetyl-CoA-acetyl transferase
NM_145942.2	1.9	3-hydroxy-3-methylglutaryl-CoenzymeA-synthase (*Hmgcs1*)
BI246566.1	1.9	hepatic lipase
NM_026784.1	1.9	phosphomevalonate kinase
BM945729.1	1.9	NAD(P) dependent steroid dehydrogenase-like
NM_011485.3	1.9	steroidogenic acute regulatory protein (*Star*)
NM_053245.1	1.9	aryl hydrocarbon receptor-interacting protein-like 1
NM_030210.1	2	acetoacetyl-CoA synthetase
NM_008288.1	2	hydroxysteroid 11-beta dehydrogenase 1 (*Hsd11b1*)
NM_025436.1	2	sterol-C4-methyl oxidase-like (*Sc4mol*)
BY616448.1	2.1	fibroblast growth factor 12
NM_138656.1	2.1	mevalonate (diphospho) decarboxylase (*Mvd*)
NM_146006.1	2.2	lanosterol synthase
NM_010742.1	2.3	lymphocyte antigen 6 complex, locus D
NM_009714.1	2.4	asialoglycoprotein receptor 1 (*Asgr1*)
NM_020010.1	2.5	cytochrome P450, 51 (*Cyp51*)
NM_145360.1	2.6	isopentenyl-diphosphate delta isomerase (*Idd1*)
NM_009731.1	5.6	aldo-keto reductase family 1, member B7 (*Akr1b7*)
NM_008493.3	6.5	leptin
*NM_015770.2*	*350*	**agouti**

Genes structurally associated with the LY mutation are shown in bold. N = 3 animals per group.

**Figure 2:**
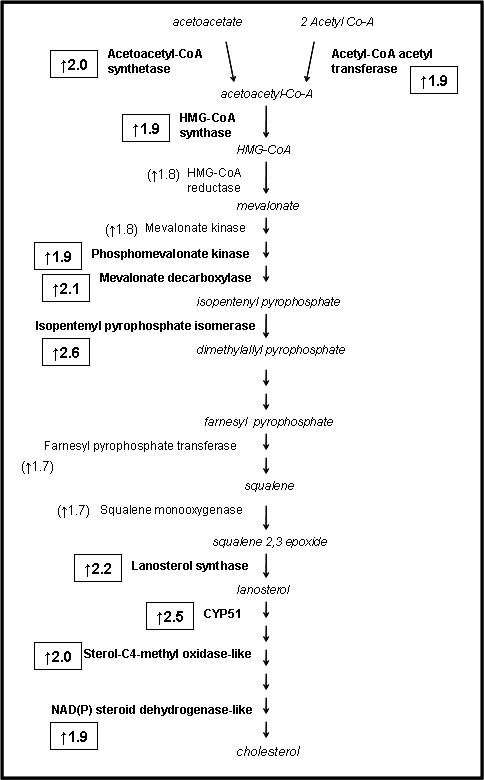
**Genes (in bold type) involved in cholesterol biosynthesis exhibiting elevated expression (p < 0.05) in 180-day LY mice as compared to black mice**. Numbers in boxes indicate fold difference (RU) in gene expression compared to black mice. Other genes in the pathway tended to be elevated (normal type with fold difference in parentheses).

Several genes involved in steroid synthesis and metabolism were up-regulated in LY mice, including steroidogenic acute regulatory protein (*Star*) and aldo-keto reductase family 1, member B7 (Akr1b7). Notably, aged LY mice had two-fold greater expression of 11beta-hydroxysteroid dehydrogenase type 1 (*Hsd11b1*) and a two-fold lesser expression of 11beta-hydroxysteroid dehydrogenase type 2 (*Hsd11b2*). Numerous differentially expressed genes are involved in cholesterol biosynthesis, e.g. isopentenyl-diphosphate delta isomerase (Idd1), Cyp51, lanosterol synthase, mevalonate (diphospho) decarboxylase, and sterol-C4-methyl oxidase-like (Sc4mol). In each case, LY mice exhibited an approximately 2-fold greater expression than black mice. Further examination of the microarray data revealed that genes representing nearly every step in the cholesterol biosynthetic pathway were expressed at a significantly higher level in LY mice (Figure 2). Other differentially expressed genes included angiotensinogen, leptin, and fibroblast growth factor 12.

**Figure 3:**
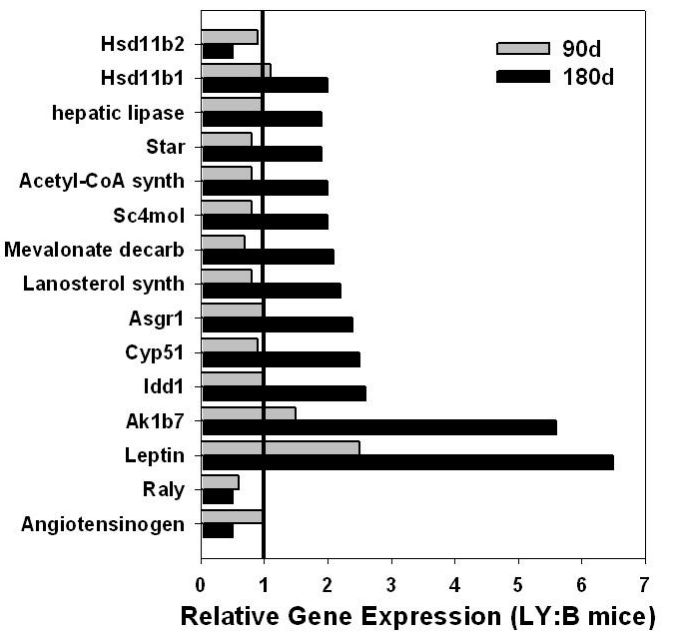
**Ovarian gene expression in 90- and 180-day LY mice relative to black mice analyzed by microarray (n = 3 for each group)**. Solid line indicates 1:1 expression ratio.

Subsequently DNA microarray experiments were performed using 90-day old LY and black mice in the same manner as described to determine whether the gene expression differences in 180-day old mice were evident in younger mice that exhibited no difference in body weight. Expression levels of agouti and *Raly *served as internal controls, (agouti: 350- and 330-fold, and *Raly*: 0.5- and 0.5-fold, 180-day versus 90-day, respectively), confirming the comparability of the two sets of microarray data. Other than agouti and *Raly*, of the genes with differential expression in 180-day old mice, only leptin showed a significant difference in 90-day old mice (Figure 3). However, leptin expression was only 2.5-fold greater in 90-day old LY mice, as compared to 6.5-fold greater in 180-day old LY mice. None of the genes involved in sterol synthesis and metabolism that were elevated in 180-day old LY mice were differently expressed in 90-day old mice. However, there were other genes that differed in expression between 90-day old LY and black mice that did not differ in aged mice. These data will be presented in a separate communication.

**Figure 4:**
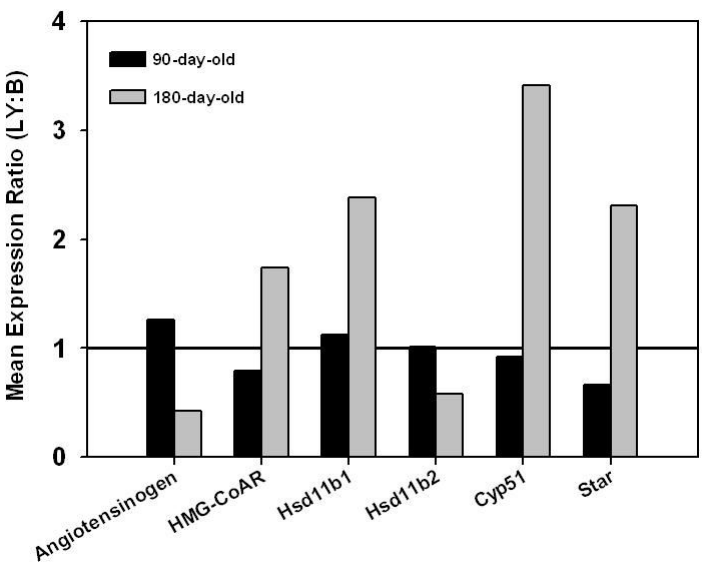
**Ovarian expression of selected genes in 90- and 180-day LY and black mice analyzed by Real Time RT-PCR**. Bars represent mean ratios (LY:black) of expression in 90- (black bars) and 180-day (gray bars) old mice. All samples were run in duplicate (n = 3 for each group).

To confirm DNA microarray data, gene expression of selected genes, i.e. angiotensinogen, *Cyp51*, 3-hydroxy-3-methylglutaryl-Coenzyme A-reductase (HMG-CoA reductase) (*Hmgcr*), *Star*, *Hsd11b1 *and *Hsd11b2*, was determined by Real time RT-PCR. Relative expression of these genes as demonstrated by RT-PCR was very similar to microarray results (Figure 4).

**Figure 5:**
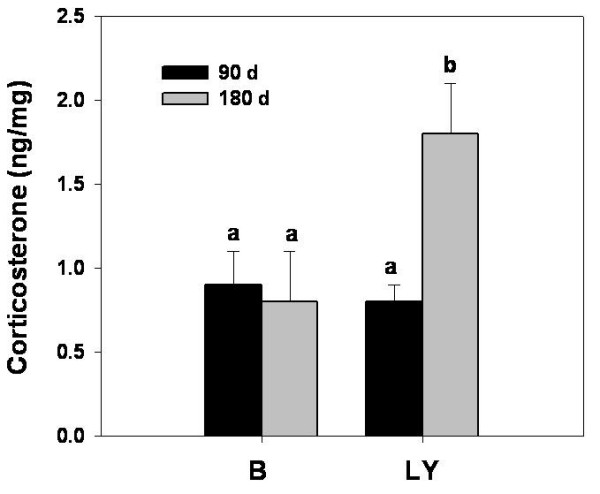
**Corticosterone concentrations in whole ovarian homogenates from 90- (black bars) and 180-day (gray bars) old black and LY mice**. Bars represent mean ± SEM (n = 5 mice per group). Different letters denote statistical significance (P < 0.05) by ANOVA with Fisher's LSD test.

*Hsd11b1 *and *Hsd11b2 *were of particular interest due to the opposing action of their protein products and divergence in their expression in obese LY mice. These enzymes interconvert corticosterone to its inactive metabolite 11-dehydrocorticosterone. RIA analysis of ovarian steroid extracts showed that aged LY mice had approximately twice the amount of corticosterone present in ovarian tissue as compared to age-matched black mice and young LY and black mice (Figure 5B), consistent with the shift in enzyme expression.

## Discussion

This is the first report of differences in the levels of ovarian gene expression in an obese mouse model. The most important finding of this study is that modified gene expression in the ovaries of aging LY mice occurs as a direct consequence of acquired obesity and is not due to an altered gonadotropic state. Since all mice were GnRH-suppressed and stimulated with exogenous gonadotropin, differences in gene expression were not due to alterations in hypothalamic-pituitary control in older mice, or to differences in estrous cycle state. Stimulation of 180-day old LY mice with exogenous gonadotropin results in similar ovarian histology and leads to the same number of preovulatory follicles and ovulated oocytes as in age-matched black mice (unpublished data). Since progressive obesity in LY mice is accompanied by the development of insulin and leptin resistance, changes in gene expression may be related to altered metabolic state. Albeit a caveat of the present study is that only whole ovarian gene expression was determined, and therefore cellular localization cannot be determined.

The *A*^*y *^mutation is a large deletion that encompasses the promoter region of the agouti gene as well as a large portion of the coding region of the adjacent upstream *Raly *gene, which is constitutively expressed in all somatic cells [9]. The *Raly *promoter is thus brought into juxtaposition with the agouti gene resulting in the ubiquitous over-expression of agouti [9]. The similar level of expression of agouti gene in both young and aging LY mice relative to black mice serves as an intrinsic control confirming the comparability of the two sets of microarrays. Conversely, the *A*^*y *^mutation leads to a reduced expression of the *Raly *gene which was expressed at half the level of black mice in both 90- and 180-day old animals. This is predicted since the LY mice are heterozygous for the agouti mutation, i.e. they possess a single normal allele.

Other than agouti and *Raly*, leptin was the only other gene that was significantly altered in both 90- and 180-day old mice, although the difference in expression was much greater in the 180-day old obese mice than in the younger mice. Leptin is primarily produced by adipocytes, and circulating leptin levels increase dramatically in aging LY mice in proportion to body weight [12]. Leptin may also be produced by theca and granulosa cells of maturing follicles [21,22]. It has been proposed that leptin resistance develops in the ovaries of obese animals [12], and increasing ovarian leptin production in obese mice may be related. Although great care was taken to remove all adhering fat tissue from the ovaries before RNA extraction, the possibility that adherant fat may be the source of the disparate leptin gene expression cannot be excluded.

A major finding of this study was the consistent enhanced ovarian expression of genes involved in cholesterol biosynthesis in obese LY mice. Aging LY mice become insulin-resistant and hyperleptinemic with increasing obesity [10,11]. It's been long recognized that hepatic cholesterol synthesis is elevated in obesity [23], and is exacerbated in diabetes [24]. Moreover, adipokines such as leptin, play a regulatory role in cholesterol metabolism. Cholesterol biosynthetic enzymes were among the hepatic genes whose expression was reduced by leptin in *ob/ob *mice [25]. Hepatic HMG-CoA-reductase activity was elevated in obese Zucker rats, which are resistant to leptin, but leptin infusion reduced HMG-CoA-reductase activity in both lean and obese rats [26]. Elevated cholesterol synthetic enzymes in the face of high leptin levels is consistent with a state of leptin resistance in the ovaries of obese LY mice.

Collectively, greater expression of cholesterol synthetic genes would suggest enhanced ovarian steroid production. Other than the glucocorticoid measurements described, ovarian extracts were insufficient to further assess steroid production in the current study. However, naturally-cycling 120- and 180-day old LY mice six days post-mating had higher intraovarian progesterone concentrations than black counterparts [Diggins and Brannian, unpublished data]. The enhanced gene expression of Akr1b7, whose protein product is an enzyme that metabolizes isocaproaldehyde, a by-product of pregnenolone synthesis, further implies an augmentation of steroid synthesis in the ovaries of obese LY mice.

One cholesterol synthetic gene over-expressed in obese LY mice that is of particular interest is *Cyp51*. *Cyp51 *catalyzes an intermediate step in the conversion of lanosterol to cholesterol, and is highly expressed in ovary and testis [27]. Specifically *Cyp51 *is responsible for the C14-demethylation of lanosterol. Regulation of *Cyp51 *expression in the gonads is gonadotropin-dependent [27,28]. Unlike other cholesterol synthetic genes, the promoter region of the *Cyp51 *gene contains both steroid- (SRE) and cAMP-response elements (CRE) [27]. The product of this reaction has been identified as meiosis-activating steroid (MAS), which induces resumption of meiosis in cumulus-enclosed oocytes [29]. In eCG-stimulated rats, *Cyp51 *expression and MAS concentrations increased in preovulatory follicles, and further increased after hCG administration [28]. Although insulin plays a critical role in regulation of hepatic *Cyp51 *expression [30], it does not appear to regulate ovarian *Cyp51 *expression [28].

Not only was there greater expression of genes involved in cholesterol synthesis, but the expression of other genes related to sterol metabolism were also elevated in obese LY mice, e.g. hepatic lipase, *Star*, and Akr1b7, also known as mouse vas deferens protein (MVDP). Hepatic lipase, Star, and Akr1b7 are all gonadotropin-regulated genes in the ovary [31-33]. Furthermore, hepatic lipase and Star expression can be modulated by insulin [34,35] and leptin [34,36]. The hyperinsulinemia/insulin-resistance of the obese LY mice may contribute to the elevated expression of these genes. Hepatic lipase is elevated in diabetics [34], and leptin enhanced hepatic lipase expression when given to ob/ob mice [25]. Star expression was increased in theca cells from follicles of women with PCOS, a syndrome characterized by hyperinsulinemia/insulin resistance [37]. Moreover, leptin bi-phasically modulates granulosa cell Star expression [36].

An interesting and unexpected finding was the reciprocal shift in *Hsd11b1 *and *Hsd11b2 *expression in aging obese LY mice. These enzymes catalyze the interconversion of bioactive and bio-inactive glucocorticoids, which is an important mechanism of regulating glucocorticoid action in many target tissues. In rodents, the major bioactive glucocorticoid is corticosterone, which is converted to inactive 11-dehydrocorticosterone by 11beta-hydroxysteroid dehydrogenase type 2 [38]. Conversely, 11-dehydro-corticosterone is converted to corticosterone by 11beta-hydroxysteroid dehydrogenase type 1. In humans, cortisol and cortisone are the major active and inactive forms, respectively. Glucocorticoids are important in the pathogenesis of obesity and insulin resistance, and expression and activity of 11beta-hydroxysteroid dehydrogenases can be altered in obesity and diabetes in a tissue-specific manner [39,40]. For example, 11beta-hydroxysteroid dehydrogenase type 1 activity was enhanced in obese rat [41] and human [39] adipose tissue, but reduced in liver. An increase in type 1 and a decrease in type 2 in the ovaries of obese LY mice would predict an overall increase in ovarian corticosterone as observed. Although the ovary does not synthesize glucocorticoids *de novo*, modulation of glucocorticoid action by interconversion of corticosterone and 11-dehydrocorticosterone likely plays an important role in regulating ovarian function. That glucocorticoids alter ovarian steroidogenesis has long been recognized [42]. Furthermore, an up-regulation of *Hsd11b1 *and down regulation of *Hsd11b2 *occurs in response to gonadotropins, particularly as associated with the LH surge [43-45]. The shift in 11beta-hydroxysteroid dehydrogenase activity leads to an increase in the ratio of active to inactive glucocortiocoid around the time of ovulation [46]. Interestingly, a higher cortisol:cortisone ratio is associated with a higher clinical pregnancy rate in IVF patients [47-49]. In addition, 11beta-hydroxysteroid dehydrogenases may be important in ovarian metabolism of mineralocorticoids [45], progestins [50], and androgens [51], which may alter ovarian function.

## Conclusion

Altered ovarian gene expression in aging LY mice is directly related to progressive obesity and is not due to an altered gonadotropic state. There was a universal up-regulation of major genes of the cholesterol synthetic pathway, as well as certain key genes involved in steroid synthesis and metabolism. Notably, obesity was associated with a regulatory shift in ovarian glucocorticoid metabolism. These results suggest that obesity impacts reproductive function in LY mice at least partly via direct modification of ovarian gene expression. Modulation of ovarian gene expression may involve altered insulin and/or leptin exposure or sensitivity, which is closely related to progressive obesity. The mechanisms by which the altered ovarian gene expression observed in obese mice affects ovarian function and fertility remains to be elucidated.

## Competing interests

The authors declare that they have no competing interests.

## Authors' contributions

JB and MD conceived and designed the study. MG, CH, and KT carried out the treatments and tissue collection, prepared preliminary data summaries, and participated in microarray analyses. KE performed RNA extractions and microarray analyses, and performed statistical analyses on microarray data. JB performed final data analysis and drafted the manuscript. All authors read and approved the final manuscript.
